# Assessing disparities in health and living conditions: a comparative study of Hungarian-speaking Roma and non-Roma women across Hungary, Romania, and Slovakia

**DOI:** 10.3389/fpubh.2024.1438018

**Published:** 2024-08-21

**Authors:** Noémi Mózes, Johanna Takács, Zoltan Ungvari, Helga Judit Feith

**Affiliations:** ^1^Department of Public Health, Faculty of Medicine, Semmelweis University, Budapest, Hungary; ^2^Department of Social Sciences, Faculty of Health Sciences, Semmelweis University, Budapest, Hungary; ^3^Vascular Cognitive Impairment, Neurodegeneration, and Healthy Brain Aging Program, Department of Neurosurgery, University of Oklahoma Health Sciences Center, Oklahoma City, OK, United States; ^4^Oklahoma Center for Geroscience and Healthy Brain Aging, University of Oklahoma Health Sciences Center, Oklahoma City, OK, United States; ^5^International Training Program in Geroscience, Doctoral School of Basic and Translational Medicine, Department of Public Health, Semmelweis University, Budapest, Hungary; ^6^Department of Health Promotion Sciences, College of Public Health, University of Oklahoma Health Sciences Center, Oklahoma City, OK, United States

**Keywords:** living condition, Roma women, health, comfort level, vulnerable groups, inequalities

## Abstract

**Background:**

The Roma minority, Europe’s largest ethnic minority, experiences significant disparities in living conditions and health outcomes compared to the non-Roma populations across the continent. Despite extensive documentation of the socio-economic challenges faced by the Roma, there is a notable lack of comparative research.

**Methods:**

This study aims to fill this gap by examining the differences in socio-economic characteristics, living conditions, and self-reported health status between Roma (R) and non-Roma (nR) women in in Hungary (HU), Romania (RO), and Slovakia (SK), providing a cross-country comparative analysis. Utilizing simple and multiple binary logistic models, our research analysed data collected from September 2020 to March 2022, involving 322 Roma and 294 non-Roma women in Hungary, 258 Roma and 183 non-Roma women in Romania, and 146 Roma and 163 non-Roma women in Slovakia.

**Results:**

Findings indicate significant associations between increased age (R:OR = 1.04[1.02,1.06], *p* < 0.001), (nR:OR = 1.04[1.02,1.05], *p* < 0.001) lower financial situation (R:OR = 2.05[1.01,4.18], *p* = 0.048) (nR:OR = 1.67[1.01,2.77], *p* = 0.047), and basic education level (R:OR = 3.60[1.29,10.08], *p* = 0.015) (nR:OR = 3.64[1.77,7.51], *p* < 0.001) with the likelihood of poor health status across both groups in Hungary. In Romania, increased age (OR = 1.04[1.02,1.06], *p* < 0.001) and basic education level (OR = 5.24[2.29,11.99], *p* < 0.001) were particularly predictive of poor health among non-Roma, while in Slovakia, age (OR = 1.05[1.02,1.07], *p* < 0.001) was a significant factor for Roma, and intermediate education level (OR = 2.68[1.16,6.20], *p* = 0.021) was for non-Roma. The study also found that a higher number of children (HU:OR = 1.35[1.12,1.63], *p* = 0.002), (RO:OR = 1.57[1.25,1.96], *p* < 0.001) and problems with housing comfort (RO:OR = 4.83[2.19,10.62], *p* = 0.015) and wall conditions (RO:OR = 2.81[1.22,6.46], *p* < 0.001) significantly impacted the health status of non-Roma women in Hungary and Romania. Conversely, an increase in household size was associated with a better health status among Roma women in Hungary (OR = 0.88[0.79,0.99]) and Slovakia (OR = 0.78[0.61,0.99]).

**Conclusion:**

By offering a novel comparative analysis, this study highlights the critical need for focused attention on the health disparities faced by Roma women, particularly those in a multiply disadvantaged situation due to their ethnic and socio-economic status.

## 1 Introduction

The Roma are one of the largest and most marginalized ethnic minorities in the European Union (EU), predominantly residing in Southeast Europe (1, 2). Historical migrations from northern India in the 11th century have led to their primary settlements in what are now Romania, Hungary, and Slovakia (3, 4). Romania hosts the largest Roma population within the EU, estimated at approximately 1.85 million. Significant Roma communities are also found in Hungary (700,000) and Slovakia (500,000) (5–7), with a noteworthy presence of Hungarian-speaking Roma in Romania (105,000) and Slovakia (80,000), representing significant proportions of the Hungarian-speaking populations in these countries (8–10).

Despite their considerable demographic presence, a large portion of the Roma population faces persistent discrimination, living in segregated conditions marked by inadequate housing and limited access to basic services (11–15). This has led to high unemployment rates and poverty levels, with around 80% living below their country’s poverty threshold and a substantial fraction lacking access to water pipelines (16–18). Health disparities are significant, with the Roma experiencing a higher prevalence of communicable diseases due to poor living conditions, further exacerbated by limited access to water and sanitation facilities (19–29). Interventions related to water, sanitation, and hygiene have been shown to substantially reduce disease incidence (30), as seen in France where improved sanitation facilities led to a notable decrease in diarrheal diseases among Roma children (31).

In Hungary, Romania, and Slovakia, the living and health conditions of the Roma share commonalities, particularly regarding inadequate infrastructure and the consequent health implications, including increased risks of respiratory and infectious diseases (26, 32–44). Moreover, the socio-economic status and unhealthy lifestyles of the Roma are strongly correlated, with significant disparities in self-reported health status observed among the Roma in comparison to non-Roma populations (45–48). In order to improve poor health, a health mediator programme was piloted in all three countries studied. Mediators have made significant progress in improving the poor health conditions in Roma settlements (49–52).

Although research often focuses on the Roma population in Central and Eastern Europe, it is worthwhile to look beyond Europe, as Roma communities live as minorities in many countries worldwide. The Roma in America, although less studied compared to their European counterparts, also face significant discrimination and marginalization (53). A survey conducted in Turkey revealed that the Roma believe poor housing conditions have a devastating impact on their health. They report that their homes are small, old, and in a state of disrepair. Additionally, many lack essential utilities such as water, electricity, and sanitation services (54). A survey conducted among women highlighted deficiencies in contraceptive use. Contributing factors to these observed disparities include socio-economic problems such as low educational attainment and the adherence to traditional customs among Roma living in settlements (55).

Members of the Roma population especially women in the diaspora are considered to be cumulatively deprived due to a combination of factors including discrimination, language difficulties, socio-economic disadvantages, and barriers to accessing education and healthcare—Language difficulties significantly contribute to the deprivation experienced by the Roma. Many Roma people speak Romani, which is often not recognized or supported by educational and governmental institutions. This lack of linguistic support can impede Roma children’s educational progress and limit their ability to access services and employment opportunities that require proficiency in the dominant language of the country they reside in (56, 57). Cultural practices and structural barriers also play an important role. Traditional Roma lifestyles and values sometimes clash with those of the host society, creating additional obstacles to integration. Moreover, institutional racism and lack of political representation hinder Roma communities’ ability to advocate for their rights and access resources necessary for their development and inclusion (58, 59).

There are significant differences between the roles of Roma men and women, especially in traditional families. Living separately from the majority society and according to their own value system, men assume the role of breadwinners. Women are responsible for washing, cooking, cleaning, and taking care of the children and the family’s health protection. They are the ones who maintain contact with healthcare professionals and support family members in seeking medical care. Furthermore, their relationships with their children and family members are very strong and intimate, making Roma women good targets for health promotion programs (60, 61).

Given the multi-layered disadvantages faced by the Roma due to their ethnic background (18, 30, 41, 62), national minority status (42, 63), and sex (16, 60), particularly among women with many children (64), This research aims to gain a better understanding of the circumstances faced by this minority, providing a basis for the introduction of further health-promoting measures.

**Figure 1:**
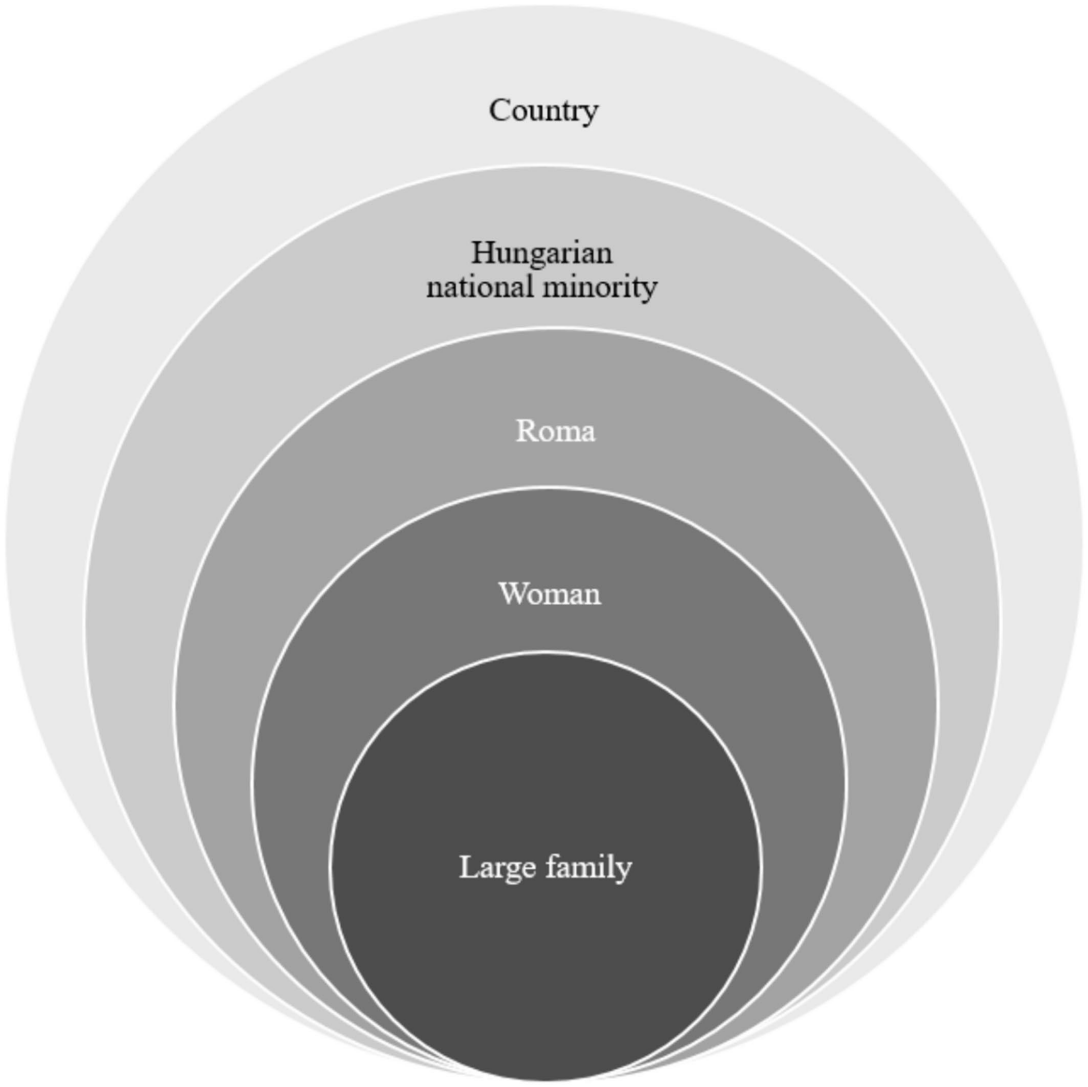
The multiple disadvantages of the Hungarian-speaking Roma. Being a female member of this national minority is particularly difficult and vulnerable, especially when living in poor conditions with many children.

This research concept builds upon studies addressing Roma inequalities (11–29). The key variables include social status, living conditions, and self-reported health. We aimed to examine these variables from two perspectives. One is the Roma and non-Roma perspective, which has already been explored. The other perspective involves comparing these variables among Roma populations in three socio-culturally distinct countries (formerly part of Hungary, where Hungarian-speaking Roma live). Our survey introduces a theoretical framework aimed at examining the living conditions and health status of the Roma population, with a special focus on the vulnerability of Roma women in Hungary, Romania, and Slovakia. This study is novel in its comprehensive assessment of living and housing conditions, health problems, and family situations using a uniform research methodology across different countries but within the same ethnicity, specifically targeting people from multiple disadvantaged minorities. It aims to illuminate the association between poor housing conditions and health issues, considering the impact of large family sizes on living standards a Figure 1 illustrates the challenges faced by the studied Hungarian-speaking female Roma population within the context of a country.

## 2 Materials and methods

### 2.1 Participants and data collection

This cross-national study was carried out in Hungary, Romania, and Slovakia from September 2020 to March 2022, spanning 19 months (46). The extended duration was due to challenges in recruiting the rural Roma population, conducting research across three countries, and the impact of the COVID-19 pandemic (65, 66).

According to the inclusion criteria, eligible participants were over 18 years of age, spoke Hungarian, and self-identified as either Roma or non-Roma. The exclusion criteria included being under 18, deemed incompetent, or unwilling to fully complete the questionnaire. However, illiteracy did not preclude participation; interviewers assisted those unable to read or write. Detailed study information was provided to all respondents, with verbal consent obtained from those who could not read or write. Convenient sampling procedure was conducted, recruiting the sample we collaborated with organizations that had strong connections with the minority (63). This procedure adhered to the Declaration of Helsinki principles, with ethical clearance obtained from ETT TUKEB (IV/3495–4/2021/EKU). The approval date from the ethics committee in the manuscript is June 30, 2020. Participants received written and, if they had questions, verbal information about their participation in the research, they provided verbal consent to participate in this study.

The survey was available in both online and paper formats at the research sites, with no content differences between them. Participants completed the survey at designated research sites, with online respondents able to ask questions of the interviewer. In the online format, respondents had to answer each question before proceeding to the next. Overall, 39.6% completed the survey online, while 60.4% used paper forms. Trained interviewers assisted participants as necessary, ensuring minimal impact on data validity. The number of non-Roma participants in each region was equivalent to the number of Roma individuals interviewed in the same region. The Slovak and Romanian samples were sourced from historical Hungarian territories and comprised individuals who self-identified as Hungarian-speaking Roma or Hungarian-speaking non-Roma. To effectively recruit participants from the Roma population, we partnered with organizations well-connected with the minority community. These included municipal settlements, Roma municipalities, Family Care Centres, Non-governmental Organizations, the Maltese Charity Service, the Catholic Charity, and the Reformed Church. Their assistance was instrumental in reaching our target population (46).

### 2.2 Measures

The study employed a self-compiled questionnaire to gather data on socio-economic characteristics (Table 1), living conditions (including housing type, building materials, condition of the walls, and comfort level), and subjective general health status. Comfort level was measured by a self-developed index considering eight variables measured in the interviews. A threshold for high and low comfort level was arbitrarily defined. The classification of the dwellings was based on the availability of utilities and amenities such as piped water, hot water, kitchen, sewage system, bathroom, toilet, gas, and electricity. A dwelling was considered to have a low comfort level if it had three or more deficiencies. Income levels were divided into below average, average, and above average based on net earnings per country. Health status was self-reported using a five-category scale, later dichotomized into good (excellent, very good, good) and poor (fair, poor) health (67).

**Table 1:** Socio-economic characteristics among Roma and non-Roma women in Hungary, Romania, and Slovakia.

	Hungary (*n* = 616)			Romania (*n* = 441)			Slovakia (*n* = 309)		
	Roma (*n* = 322)	Non-Roma (*n* = 294)	*p*[ES]	Roma (*n* = 258)	Non-Roma (*n* = 183)	*p*[ES]	Roma (*n* = 146)	Non-Roma (*n* = 163)	*p*[ES]
Age, M ± SD	44.70 ± 13.99	46.29 ± 15.07	0.174[g = 0.11]	39.37 ± 14.25	40.6 ± 16.64	0.406[g = 0.08]	39.32 ± 15.0	39.67 ± 14.19	0.833[g = 0.02]
Education, frequency (%)									
Primary school	194 (60.2)	44 (15.0)	<0.001 [V = 0.56]	210 (81.4)	53 (29.0)	<0.001 [V = 0.64]	89 (61.0)	9 (5.5)	<0.001 [V = 0.70]
Apprenticeship/vocational training	64 (19.9)	33 (11.2)		38 (14.7)	18 (9.8)		31 (21.2)	21 (12.9)	
High school	41 (12.7)	87 (29.6)		9 (3.5)	48 (26.2)		23 (15.8)	52 (31.9)	
College/university	23 (7.1)	130 (44.2)		1 (0.4)	64 (35.0)		3 (2.1)	81 (49.7)	
Habitation									
Chief town of a county	65 (20.2)	60 (20.4)	0.008 [V = 0.13]	42 (16.3)	50 (27.3)	<0.001 [V = 0.24]	51 (34.9)	55 (33.7)	0.150 [V = 0.11]
Town	129 (40.2)	150 (51.0)		30 (11.6)	43 (23.5)		24 (16.4)	41 (25.2)	
Village/hamlet	128 (39.8)	84 (28.6)		186 (72.1)	90 (49.2)		71 (48.6)	67 (41.1)	
Financial situation									
Below average	281 (87.3)	141 (48.0)	<0.001 [V = 0.42]	245 (95.0)	124 (67.8)	<0.001 [V = 0.38]	99 (67.8)	146 (31.9)	<0.001 [V = 0.36]
Average	38 (11.8)	134 (45.6)		13 (5.0)	34 (18.6)		32 (21.9)	50 (46.6)	
Above average	3 (0.9)	19 (6.5)		0 (0)	25 (13.7)		15 (10.3)	74 (21.5)	
Marital status									
Single	55 (17.1)	52 (17.7)	0.072 [V = 0.12]	48 (18.6)	44 (24.0)	0.001 [V = 0.21]	26 (17.8)	31 (19.0)	0.382 [V = 0.12]
Married	105 (32.6)	114 (38.8)		95 (36.8)	84 (45.9)		58 (39.7)	78 (47.9)	
Partnership	79 (24.5)	47 (16.0)		84 (32.6)	27 (14.8)		45 (30.8)	36 (22.1)	
Divorced	42 (13.0)	48 (16.3)		9 (3.5)	11 (6.0)		9 (6.2)	7 (4.3)	
Widowed	41 (12.7)	33 (11.2)		22 (8.5)	17 (9.3)		8 (5.5)	11 (6.7)	
Household composition									
Single occupancy	51 (15.8)	65 (22.1)	<0.001 [V = 0.23]	16 (6.2)	22 (12.0)	<0.001 [V = 0.28]	15 (10.3)	20 (12.3)	0.698 [V = 0.10]
Married/cohabiting with no dependent children	48 (14.9)	85 (28.8)		33 (12.8)	35 (19.1)		39 (26.7)	39 (23.9)	
Married/cohabiting with dependent children	131 (40.7)	82 (27.9)		105 (40.7)	60 (32.8)		53 (36.3)	66 (40.5)	
Married/cohabiting with dependent children and grandparents	22 (6.8)	11 (3.7)		52 (20.2)	13 (7.1)		9 (6.2)	7 (4.3)	
Single parent family	49 (15.2)	28 (9.5)		27 (10.5)	12 (6.6)		10 (6.8)	6 (3.7)	
Multi-person household, with parents	21 (6.5)	23 (7.8)		25 (9.7)	41 (22.4)		20 (13.7)	25 (15.3)	
Number of children, M ± SD	2.56 (1.82)	1.64 (1.38)	<0.001[g = 0.57]	2.99 (2.00)	1.50 (1.53)	<0.001[g = 0.82]	1.79 (2.00)	1.29 (1.10)	0.006[g = 0.31]
Number of members in the household, M ± SD	3.76 (2.27)	2.69 (1.45)	<0.001[g = 0.56]	5.12 (2.81)	3.26 (1.52)	<0.001[g = 0.79]	3.42 (1.97)	3.17 (1.40)	0.189[g = 0.15]

[ES], effect size; g, Hedges’ g; V, Cramer’s V.

### 2.3 Data analysis

Descriptive statistics and frequency distributions described the sample’s socio-economic and living conditions. Independent samples t-tests with Hedges’s g and one-way ANOVA with omega-squared measured differences between and within countries and ethnic groups. Cross-tabulations and Pearson’s chi-square test assessed the association between ethnicity and variables, with significance set at *p* < 0.05. IBM SPSS Statistics for Windows, Version 25.0, and R corrplot package (68) facilitated statistical analysis and visualization. Simple and multiple binary logistic models determined the impact of socio-economic and living conditions on health status by country and ethnicity.

## 3 Results

### 3.1 Study sample

Our study encompassed 1,366 female participants from Hungary, Romania, and Slovakia, distributed as follows: Hungary-Roma: 322, Hungary-non-Roma: 294, Romania-Roma: 258, Romania-non-Roma: 183, Slovakia-Roma: 146, Slovakia-non-Roma: 163 (Table 1).

### 3.2 Socio-economic characteristics of Roma and non-Roma women

We evaluated the socio-economic status and living conditions among Roma and non-Roma women across the three countries (Table 1). Age differences between Roma and non-Roma women across the countries were not significant. However, Hungarian participants (M = 45.46, SD = 14.53) were significantly older than those from Romania (M = 39.88, SD = 15.28) and Slovakia (M = 39.51, SD = 14.62) (*F*(2,1363) = 25.693, *p* < 0.001, ω2 = 0.03). Roma women consistently exhibited lower educational achievements compared to their non-Roma counterparts, with the disparity most pronounced in Romania, where approximately 80% of Roma women attained only primary school education. Financially, a larger percentage of Roma women reported below-average financial situations: 87.3% in Hungary, 95.0% in Romania, and 67.8% in Slovakia. Marital status and ethnicity correlations were significant in Romania, with a higher percentage of Roma women living in partnerships. Furthermore, household composition significantly differed in Hungary and Romania, with Roma women more likely to reside in multi-person households including dependent children and grandparents.

Roma women in Hungary, Romania, and Slovakia consistently have larger families compared to their non-Roma counterparts. Specifically, 30.6% of Roma women (*n* = 222) have three to four children, and 13.8% (*n* = 100) have five or more children. In contrast, among non-Roma women, 15.9% (*n* = 102) have three to four children, and only 3.2% (*n* = 20) have five or more. This pattern suggests a higher number of individuals living in Roma households than in non-Roma ones across all three countries.

### 3.3 Living conditions among Roma and non-Roma women

Investigating housing conditions revealed significant ethnic disparities (Table 2). While nearly half of the Roma women reside in family houses across the studied countries, a disproportionately higher percentage of them live in temporary shelters when compared to their non-Roma counterparts. This trend is most pronounced among Roma women in Romania, where the incidence of residing in temporary housing is notably higher.

**Table 2:** Living conditions among Roma and non-Roma women in Hungary, Romania, and Slovakia.

	Hungary (*n* = 616)			Romania (*n* = 441)			Slovakia (*n* = 309)		
	Roma (*n* = 322)	Non-Roma (*n* = 294)	*p*[ES]	Roma (*n* = 258)	Non-Roma (*n* = 183)	*p*[ES]	Roma (*n* = 146)	Non-Roma (*n* = 163)	*p*[ES]
Housing type, frequency (%)									
Detached house	222 (68.9)	185 (62.9)	<0.001 [V = 0.25]	136 (52.7)	121 (66.1)	NA	92 (63)	117 (71.8)	NA
Terraced house	36 (11.2)	25 (8.5)		2 (0.8)	10 (5.5)		10 (6.8)	17 (10.4)	
Block of flat	24 (7.5)	69 (23.5)		37 (14.3)	36 (19.7)		32 (21.9)	25 (15.3)	
Farmhouse	24 (7.5)	6 (2)		39 (15.1)	14 (7.7)		8 (5.5)	4 (2.5)	
Temporary shelter	16 (5)	9 (3.1)		44 (17.1)	2 (1.1)		4 (2.7)	0 (0)	
Building/walls materials									
Brick	220 (68.3)	221 (75.2)	<0.001 [V = 0.18]	101 (39.1)	107 (58.5)	<0.001 [V = 0.23]	92 (63)	130 (79.8)	0.001 [V = 0.22]
Concrete plates	24 (7.5)	39 (13.3)		22 (8.5)	23 (12.6)		26 (17.8)	23 (14.1)	
Cob wall	78 (24.2)	34 (11.6)		135 (52.3)	53 (29)		28 (19.2)	10 (6.1)	
Condition of walls									
No problem	221 (68.6)	255 (86.7)	<0.001 [Φ = 0.22]	102 (39.5)	156 (85.2)	<0.001 [Φ = 0.46]	92 (63)	152 (93.3)	< 0.001 [Φ = 0.37]
Problem	101 (31.4)	39 (13.3)		156 (60.5)	27 (14.8)		54 (37)	11 (6.7)	
Comfort level									
Low	62 (19.3)	13 (4.4)	<0.001 [Φ = 0.23]	186 (72.1)	34 (18.6)	<0.001 [Φ = 0.53]	46 (31.5)	7 (4.3)	<0.001 [Φ = 0.36]
High	260 (80.7)	281 (95.6)		72 (27.9)	149 (81.4)		100 (68.5)	156 (95.7)	

[ES], effect size; V, Cramer’s V; Φ, Phi; NA, due to the small cells size, the assumption of Pearson’s chi-square test was violated, *p*[ES] not calculated.

The study identified a notable link between housing material quality and ethnicity across Hungary, Romania, and Slovakia. In Hungary and Slovakia, the majority of participants, regardless of ethnicity, reside in brick constructions. However, a significant proportion of Roma women—approximately a quarter in Hungary and one-fifth in Slovakia—live in houses or apartments made from cob, a ratio that starkly contrasts with that of non-Roma women. The situation is more acute in Romania, where over half of Roma women inhabit cob wall residences, a condition also experienced by nearly one-third of non-Roma women, highlighting a broader issue of housing quality in the region.

Additionally, the condition of housing walls—specifically issues with dampness and mold—significantly correlates with ethnicity across all countries. Roma women are disproportionately affected by these problems, particularly in Romania, indicating a higher prevalence of substandard living conditions within this community.

The overall comfort level of housing also displays a significant ethnic disparity. Roma women are more likely to experience lower housing comfort levels than their non-Roma counterparts in all three countries. This disparity is most pronounced in Romania, where a substantial majority of Roma women reside in low-comfort level housing, compared to one-fifth in Hungary and nearly one-third in Slovakia.

### 3.4 Subjective health status

The analysis revealed a significant association between ethnicity and self-reported health status among the study participants (χ^2^(1,N = 1,366) = 23.844, *p* < 0.001). A higher proportion of Roma women (40.9%, *n* = 297) reported poor health compared to non-Roma women (28.3%, *n* = 181). This trend was consistent in Hungary, where 43.8% (*n* = 141) of Roma women reported poor health versus 29.3% (*n* = 86) of non-Roma women, and in Slovakia, with 32.9% (*n* = 48) of Roma versus 19.6% (*n* = 32) of non-Roma women reporting poor health. However, in Romania, the difference between Roma (41.9%, *n* = 108) and non-Roma women (34.4%, *n* = 63) in reporting poor health was not statistically significant, indicating a somewhat narrower gap in perceived health status between the two groups in this country.

### 3.5 Simple and multiple analysis

**Figure 2:**
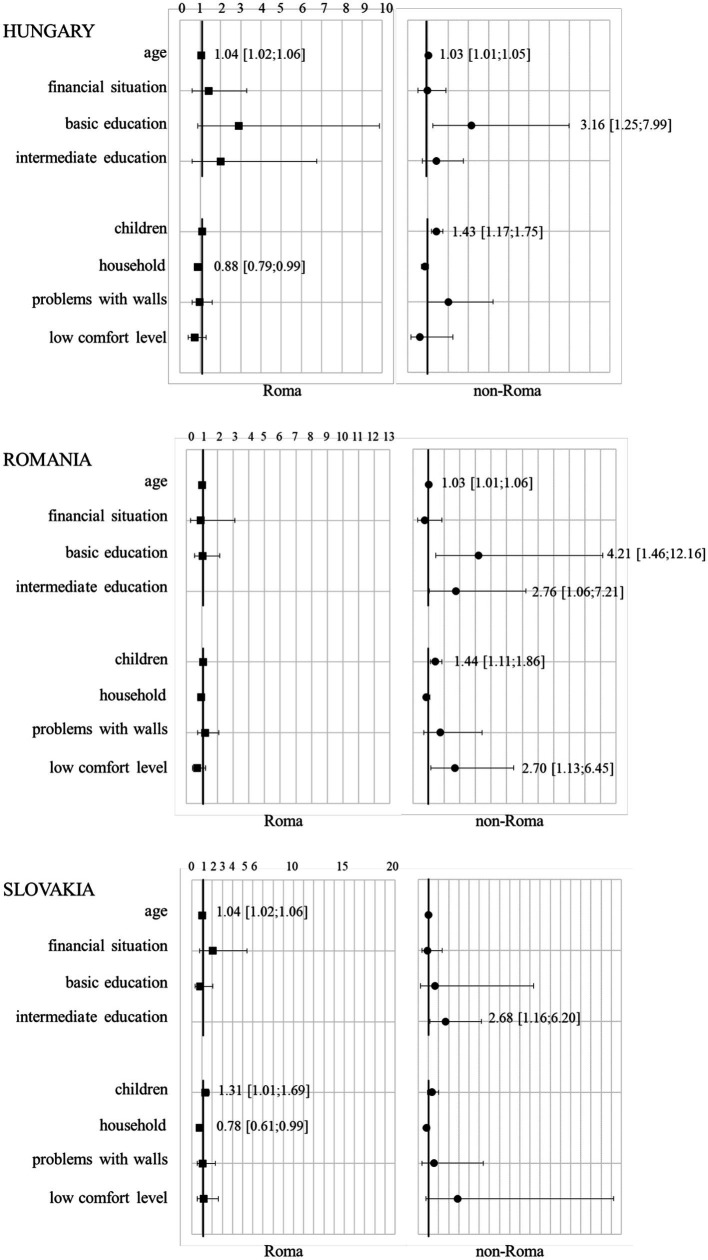
Multiple binary logistic regression analysis of the association between socio-economics and living conditions as well as subjective health status by country and ethnicity. Multiple binary logistic regression, outcome: subjective health status (good vs. poor health status), odds ratios are represented by squares (for Roma women) and circles (for non-Roma women) with 95% confidence interval, horizontal line: odds ratio 1 that indicates that the “event” (predictor of socio-economics, living conditions) is equally likely to occur in both groups of subjective health status (good vs. poor health status). Odds ratios with 95% confidence interval are indicated for significant predictors.

We analysed the relationship between socio-economic factors, age, financial status, education level, and subjective health status across the participant groups. In Hungary, factors such as increased age, a below-average financial situation, and a basic level of education were linked to a higher likelihood of reporting poor health status, regardless of ethnicity. In Romania, these associations were notably significant within the non-Roma population, with increased age and a basic level of education elevating the risk of poor health. In Slovakia, the risk factors varied with ethnicity: increased age was a significant factor for Roma women, while an intermediate level of education was associated with poor health status among non-Roma women (Table 3). Multiple analysis across the countries identified lower education levels as a critical determinant of poor health status, predominantly among non-Roma women (Figure 2).

**Table 3:** Simple binary logistic regression analysis of the association between socioeconomics and subjective health status by country and ethnicity.

	Hungary		Romania		Slovakia	
	Roma	Non-Roma	Roma	Non-Roma	Roma	Non-Roma
Socioeconomics	OR[95%CI] *p*		OR[95%CI] *p*		OR[95%CI] *p*	
Age	**1.04[1.02,1.06]** **<0.001**	**1.04[1.02,1.05]** **<0.001**	1.01[0.996,1.03] 0.139	**1.04[1.02,1.06]** **<0.001**	**1.05[1.02,1.07]** **<0.001**	1.01[0.985,1.04] 0.386
Financial situation						
Average/above average	ref	ref	ref	ref	ref	ref
Below average	**2.05[1.01,4.18]** **0.048**	**1.67[1.01,2.77]** **0.047**	1.16[0.37,3.65] 0.799	1.64[0.83,3.24] 0.153	1.95[0.89,3.24] 0.096	1.37[0.61,3.06] 0.450
Education						
Advanced	ref	ref	–	ref	–	ref
Basic	**3.60[1.29,10.08]** **0.015**	**3.64[1.77,7.51]** **<0.001**	1.21[0.63,2.31] 0.567	**5.24[2.29,11.99]** **<0.001**	1.46[0.70,3.08] 0.314	2.03[0.37,11.16] 0.416
Intermediate	2.13[0.73,6.18] 0.166	1.56[0.88,2.77] 0.127	ref	2.17[0.96,4.87] 0.061	ref	**2.68[1.16,6.20]** **0.021**

Simple binary logistic regression, outcome: subjective health status (good vs poor health status); OR, odd ratio; 95% CI, 95% confidence interval; significant predictors are in bold; ref, reference category; −, no data in the category.

The impact of living conditions on health was also explored. A higher number of children correlated with an increased likelihood of poor health status among non-Roma women in Hungary and Romania. Additionally, in Romania, problematic wall conditions and a low comfort level in housing were significantly associated with poor health among non-Roma women. Conversely, in Slovakia, these living conditions did not show a significant correlation with health status among either group (Table 4).

**Table 4:** Simple binary logistic regression analysis of the association between living conditions and subjective health status by country and ethnicity.

	Hungary		Romania		Slovakia	
	Roma	Non-Roma	Roma	Non-Roma	Roma	Non-Roma
Living conditions	OR[95%CI] *p*		OR[95%CI] *p*		OR[95%CI] *p*	
Number of children	1.04[0.92,1.18] 0.508	**1.35[1.12,1.63]** **0.002**	1.07[0.95,1.21] 0.276	**1.57[1.25,1.96]** **<0.001**	1.12[0.95,1.34] 0.183	1.20[0.85,1.70] 0.303
Number of members in the household	0.90[0.81,1.00] 0.052	0.96[0.80,1.15] 0.652	0.99[0.90,1.08] 0.734	0.96[0.78,1.17] 0.668	0.92[0.76,1.11] 0.355	0.92[0.69,1.21] 0.545
Condition of walls						
No problem	ref	ref	ref	ref	ref	ref
Problem	0.93[0.58,1.50] 0.767	1.84[0.92,3.68] 0.086	1.12[0.68,1.86] 0.661	**2.81[1.22,6.46]** **0.015**	1.18[0.58,2.40] 0.649	1.59[0.40,6.37] 0.512
Comfort level						
High	ref	ref	ref	ref	ref	ref
Low	0.77[0.44,1.36] 0.370	1.08[0.32,3.60] 0.902	0.74[0.43,1.28] 0.278	**4.83[2.19,10.64]** **<0.001**	1.31[0.63,2.72] 0.477	3.28[0.70,15.48] 0.133

Simple binary logistic regression, outcome: subjective health status (good vs. poor health status); OR, odd ratio; 95% CI, 95% confidence interval; significant predictors are in bold; ref, reference category.

Further multiple analysis underscored the influence of family size on health, with a higher number of children being associated with poor health status among non-Roma women in Hungary and Romania, and Roma women in Slovakia. In Romania, a low housing comfort level was also a predictor of poor health among non-Roma women. Meanwhile, among Roma women in Hungary and Slovakia, an increase in household size correlated with a reduced likelihood of reporting poor health status (Figure 2).

## 4 Discussion

Recent surveys have increasingly focused on the Roma minority (3, 4, 15, 17, 24), yet studies specifically addressing Roma women (16, 43, 60), particularly Hungarian-speaking Roma women, remain scarce (42, 63). This gap in the literature underscores the importance of our research, which zeroes in on a doubly marginalized group: women who are not only part of a national minority (42, 63) but also live in multiply disadvantaged conditions due to their Roma origin (18, 23–28, 32–41, 44, 62) and socio-economic circumstances (64).

Our findings resonate with previous studies highlighting the Roma population’s low educational and financial status and poor living conditions, factors known to exacerbate health disparities (10, 16–18, 31–34, 62). In alignment with findings from European Union studies, our research also demonstrates that Roma communities across all surveyed countries experience significantly inferior housing conditions. However, Roma housing and health problems are also present outside Europe (53, 54). Particularly, our study corroborates that, among the three countries analysed, Roma in Romania face the most severe deprivation regarding their living environments (16, 43, 62). This group exhibits a notably higher likelihood of encountering a confluence of utility deficiencies, underscoring the acute nature of housing inadequacies within this community.

Echoing the findings of Mózes et al., our research confirms the significantly poorer housing conditions among Roma compared to non-Roma populations in Hungary and Romania (42, 43). While extending our investigation into health status predictors in Romania, we discovered that issues like problematic walls and low housing comfort levels were notably linked to poorer health outcomes among non-Roma women. At the same time unexpectedly, there was no significant difference between the self-reported health status of Roma and non-Roma women in Romania. This can probably be explained by the fact that, compared to the non-Roma population in Hungary and Slovakia, the living conditions of the non-Roma population in Romania are closer to those of the Roma population in Romania. The condition of homes is worse, and the proportion of problematic, low-comfort, cobbled properties is higher than in the other two countries, which presumably worsens self-reported health status.

An intervention in Paris aimed at enhancing the living conditions for the Roma community presents a compelling case study (31). The establishment of mobile toilets in one settlement, accompanied by health education sessions focusing on hand hygiene, disease prevention, and sanitation practices, led to tangible improvements in women’s quality of life. Reports indicated a decrease in the avoidance of urination and reduced incidence of health issues such as diarrhoea, urinary tract infections, and eye infections among women (31). This intervention underscores the potential benefits of similar initiatives elsewhere. However, the financial barriers faced by many Roma families highlight the urgent need for external support to facilitate such basic yet critical improvements in living conditions and hygiene practices.

Consistent with existing literature, our findings confirm that self-reported health among Roma residents is generally poorer compared to non-Roma populations, a trend observed in Hungary and Slovakia (29, 46, 48). However, in Romania, we did not identify a significant correlation between ethnicity and health status, suggesting nuanced health dynamics within different national contexts (29, 46, 48).

Vokó et al. highlighted that the lower socio-economic status among Hungarian Roma living in settlements contributes significantly to their self-perceived poor health (48). Extending this analysis, our research indicates that low socio-economic status is a determinant of poor health across both Roma and non-Roma populations in all three studied countries. Notably, education emerged as a critical predictor of health status in our multiple analysis, but this association was prominent only among non-Roma women.

In Hungary, Romania, and Slovakia, a health mediator program was launched to improve the situation of the Roma population. The program aims to enhance the health knowledge of Roma communities, increase access to healthcare services, and boost participation in public health interventions. This initiative has proven to be a good practice, as it involves selecting individuals from Roma communities, providing them with education, and then having these trained mediators share their acquired knowledge with their own communities. This approach is more effective because Roma are more likely to accept advice from community members rather than outsiders. The program significantly contributes to equipping Roma communities with valid health information (49–52).

No association was found between income, education and self-reported health status among the Roma population in any country. It is assumed that this result is due to the fact that the Roma tradition of large families is a more important influence than the socio-economic status of the individual, as the rest of the analysis shows. The role of family structure in health outcomes, particularly among Roma communities, warrants special attention. The cohesive nature of Romani extended families, characterized by their supportive and compassionate care for members, suggests that a larger family size may act as a protective factor against poor health. Our findings support this hypothesis, demonstrating that in Hungary and Slovakia, an increase in household size correlates with a reduced likelihood of reporting poor health among Roma women (60). This underscores the protective value of the traditional extended family model for Roma, emphasizing the importance of social support systems in fostering health and well-being.

## 5 Strengths and limitations

Our research addresses a critical gap in the existing literature by focusing on the Hungarian-speaking Roma and non-Roma populations in Romania and Slovakia, groups that represent a “minority within a minority.” Achieving a significant recruitment of Roma respondents, alongside a comparable sample of non-Roma respondents, underscores the robustness of our methodology and the relevance of our findings, especially considering the challenges posed by the COVID-19 pandemic in reaching what is often considered the most inaccessible population. The main strength of the research is that it allowed the participation of illiterate individuals.

One of the primary limitations of our study is the inability to calculate precise response rates among Roma women, stemming from the difficulty in accurately determining the total female population within each country’s Roma community. Furthermore, the extended duration of data collection, necessitated by the pandemic’s constraints and the inherent challenges in accessing specific segments of the Roma population, may have influenced the study’s outcomes. Additional limitations include the potential for survey responses to be biased due to errors, social desirability, or recall problems, as well as the exclusion of non-Hungarian speaking individuals. Considering the selection bias, our results cannot be generalized to the Roma and non-Roma populations.

These limitations highlight the complexities involved in conducting research within highly marginalized and transient communities and underscore the need for innovative approaches to data collection in such contexts.

## 6 Conclusion

Our research underscores the significant impact of socio-economic and housing conditions on the health status of Hungarian-speaking Roma and non-Roma populations. A pivotal discovery of our study is the beneficial role of the extended family structure among the Roma, where an increase in household size correlates with improved health outcomes. This suggests that the traditional value of community cohesion continues to play a vital role in enhancing individual well-being, mirroring its historical significance. Another important conclusion of our research is that although Hungarian-speaking Roma living outside the borders of Hungary belong to the same ethnic and linguistic group and share similar histories, geopolitical, and sociocultural characteristics, belonging to the same country is a strong predictor.

Given the scarcity of research on Hungarian-speaking Roma and non-Roma communities, future expansions of this study to include broader participant groups from neighbouring countries are essential. Our findings contribute to a deeper understanding of the unique challenges faced by Hungarian-speaking Roma living outside Hungary, especially women who navigate the complexities of dual identity and gender discrimination. Recognizing the pivotal role women play in maintaining family health, it becomes imperative to develop policies that address the specific health needs of these populations, taking into account their living conditions and socio-economic status.

In conclusion, our study not only highlights the pressing health disparities faced by Hungarian-speaking Roma and non-Roma populations but also emphasizes the protective effect of familial and community support systems. To mitigate these disparities, targeted research and policy interventions are necessary, focusing on improving living conditions, access to education, and overall socio-economic development.

## Data Availability

The raw data supporting the conclusions of this article will be made available by the authors, without undue reservation.
